# Is Osteopathic Manipulative Treatment Clinically Superior to Sham or Placebo for Patients with Neck or Low-Back Pain? A Systematic Review with Meta-Analysis

**DOI:** 10.3390/diseases12110287

**Published:** 2024-11-08

**Authors:** Luis Ceballos-Laita, Sandra Jiménez-del-Barrio, Andoni Carrasco-Uribarren, Ricardo Medrano-de-la-Fuente, Román Robles-Pérez, Edzard Ernst

**Affiliations:** 1 Grupo de Investigación Clínica en Ciencias de la Salud, Departamento de Cirugía, Oftalmología, Otorrinolaringología y Fisioterapia, Universidad de Valladolid, Campus Duques de Soria, 42004 Soria, Spain; luis.ceballos@uva.es (L.C.-L.);; 2 Departamento de Fisioterapia, Universidad Internacional de Cataluña, 08195 Sant Cugat del Valles, Spain; 3 Emeritus Professor, University of Exeter, Exeter EX4 4PY, UK; e.ernst@exeter.ac.uk

**Keywords:** osteopathy, osteopathic manipulative treatment, neck pain, low-back pain

## Abstract

**Objectives**: The aim of this systematic review and meta-analysis was to compare whether osteopathic manipulative treatment (OMT) for somatic dysfunctions was more effective than sham or placebo interventions in improving pain intensity, disability, and quality of life for patients with neck pain (NP) or low-back pain (LBP). **Methods**: A systematic review and meta-analysis was carried out. Searches were conducted in PubMed, Physiotherapy Evidence Database, Cochrane Library, and Web of Science from inception to September 2024. Studies applying a pragmatic intervention based on the diagnosis of somatic dysfunctions in patients with NP or LBP were included. The methodological quality was assessed with the PEDro scale. The quantitative synthesis was performed using random-effect meta-analysis calculating the standardized mean difference (SMD) with RevMan 5.4. The certainty of evidence was evaluated using GRADEPro. **Results**: Nine studies were included in the qualitative synthesis, and most of them showed no superior effect of OMTs compared to sham or placebo in any clinical outcome. The quantitative synthesis reported no statistically significant differences for pain intensity (SMD = −0.15; −0.38, 0.08; seven studies; 1173 patients) or disability (SMD = −0.09; −0.25, 0.08; six studies; 1153 patients). The certainty of evidence was downgraded to moderate, low, or very low. **Conclusions**: The findings of this study reveal that OMT is not superior to sham or placebo for improving pain, disability, and quality of life in patients with NP or LBP.

## 1. Introduction

Neck pain (NP) and low-back pain (LBP) are the most common causes of pain and disability in adult populations [1,2]. They affect more than 80% of people at least once in their lifetime, and their prevalence is rising in all age groups [3,4,5,6], leading to an increased demand for healthcare consultations and considerable financial burden for societies across the globe. Many patients suffering NP or LBP turn to complementary and alternative therapies, such as osteopathy [7,8,9].

Osteopathy is a holistic approach that focuses on the manual manipulation of the musculoskeletal system to restore physiological function and support homeostasis, which may be disrupted by somatic dysfunctions. This practice, commonly known as Osteopathic Manipulative Treatment (OMT), is claimed to promote overall wellness without reliance on pharmaceuticals or invasive procedures [10].

OMT uses manual techniques either for the diagnosis and for treatment of so-called somatic dysfunctions, defined as the “impaired or altered function of components of the somatic system, including skeletal, arthrodial, and myofascial structures, as well as related vascular, lymphatic, and neural elements, and it is characterized by positional asymmetry, restricted range of motion, tissue texture abnormalities, and/or tenderness” [11]. The diagnosis of somatic dysfunctions relies on the manual palpation of tissues to identify these specific characteristics. Osteopathic interventions incorporate a wide range of manual techniques to treat somatic dysfunctions, including visceral manipulation, craniosacral techniques, high-velocity low-amplitude (HVLA) adjustments, articulatory techniques, soft-tissue stretching, myofascial release, and muscle energy techniques, among others. Osteopathic interventions are applied to the entire body, regardless of the symptomatic area, either independently or in combination with other treatments.

Several systematic reviews with meta-analyses found clinical benefits from a combination of osteopathic techniques in patients with NP and LBP [12,13,14,15]. However, these reviews have methodological flaws, such as including congress abstracts, pilot studies that do not aim to evaluate clinical effectiveness, and unpublished materials from osteopathic institutions as relevant studies. These studies also combine quantitative results from studies using cranial or visceral interventions in isolation with those using pragmatic interventions, and treat different comparators (such as exercise, placebo techniques or waiting lists) as if they were equivalent. Recent systematic reviews and meta-analyses have evaluated the clinical effectiveness of craniosacral interventions and visceral manipulations in isolation either in musculoskeletal or non-musculoskeletal disorders, and both reviews concluded that cranial and visceral osteopathy is not supported by sound evidence [16,17].

Therefore, the aim of this systematic review and meta-analysis is to determine whether OMTs for so-called somatic dysfunctions are more effective than sham or placebo interventions in improving clinical outcomes for patients with NP or LBP.

## 2. Materials and Methods

### 2.1. Study Design

This systematic review and meta-analysis was carried out following the Preferred Reporting Items for Systematic reviews and Meta-Analyses (PRISMA) statement and the Cochrane recommendations for systematic reviews with meta-analyses [18]. The study protocol was pre-registered in PROSPERO under the unique identification number (CRD42024595500).

### 2.2. Search Strategy

Searches were conducted in PubMed (MEDLINE), the Physiotherapy Evidence Database (PEDro), the Cochrane Library, and Web of Science (WoS) from inception to September 2024. Medical Subject Headings (MeSH) terms and free-text keywords, including “osteopathic manipulation”, “osteopathic medicine”, “osteopathic treatment”, “osteopathic intervention”, “osteopathic manipulative treatment”, “neck pain”, and “low-back pain”, were used in the search strategy. The specific search strategy for each database is detailed in Appendix A. Additionally, the reference lists of the included studies and relevant previous systematic reviews were manually searched.

### 2.3. Eligibility Criteria

The inclusion criteria were developed following the PICOS method:

- Population: Patients with NP or LBP as diagnosed clinically.
- Intervention: Holistic approach of OMT based on the diagnosis of the somatic dysfunctions. According to the benchmarks for training in osteopathy, OMT includes articular, myofascial, cranial, and visceral techniques [10].
- Comparison: Sham, placebo, or simulated techniques.
- Outcomes: Pain intensity, disability and/or quality of life.
- Study design: Randomized clinical trials.

Studies were excluded if they met the following criteria: included healthy participants or patients with non-musculoskeletal conditions, applied osteopathic techniques in isolation or did not apply a pragmatic OMT intervention based on the diagnosis of somatic dysfunctions, reported outcome variables not related to the clinical status of the patients, or the outcome variables were not registered using validated instruments.

### 2.4. Study Selection

The reference lists obtained from each database were exported to Mendeley to eliminate duplicates. Two authors (LC and SJ) independently assessed the titles and abstracts of each study to determine their potential eligibility. Full-text reviews were conducted for the studies that met the inclusion criteria after the title and abstract screening. In the case of discrepancies, a third reviewer was consulted to resolve them (RM).

#### Data Extraction

Data extraction was carried out independently by two reviewers (LC and SJ) using a predefined sheet based on the Cochrane Collaboration guidelines. The extracted data included population characteristics (mean age, diagnosis), details of the interventions (techniques applied, session duration, number of sessions per week, and total sessions), outcome variables, and results.

### 2.5. Methodological Quality Assessment

The methodological quality of the included studies was evaluated by two independent reviewers (LC and SJ) using the PEDro scale, which is based on an 11-item checklist developed from a Delphi consensus [19,20,21]. The PEDro scale assesses the methodological rigor of clinical trials by evaluating key aspects such as randomization, allocation concealment, blinding, and statistical reporting. A score of 0–3 was deemed “poor” methodological quality, scores between 4 and 5 were classified as “fair”, scores from 6 to 8 were classified as good, and scores of 9 or above were classified as “excellent”. The first item of the PEDro scale, which assesses the specification of eligibility criteria and pertains to external validity, was not included in the total score calculation. The remaining 10 items focus on internal validity and interpretability [22].

### 2.6. Data Synthesis and Analysis

A qualitative synthesis of the results was conducted, and whenever it was possible, a quantitative synthesis (meta-analysis) was carried out using the RevMan 5.4 software.

Data were combined for meta-analysis when at least two studies were sufficiently homogeneous. Mean differences (MD), standard deviations (SD), and sample sizes at each time point were extracted for each group. If MDs were not reported and could not be calculated, the post-intervention means were used. When none of the required data were provided in the articles, the authors were contacted via email to request the missing information.

Outcomes were analyzed by calculating the standardized mean difference (SMD) due to the use of different scales and questionnaires across the included studies, with 95% coefficient intervals (CIs). SMD values were interpreted as small (SMD between 0.2 and 0.5), medium (SMD between 0.5 and 0.8), or large (SMD ≥ 0.8) [23]. Statistical significance was set at *p* value < 0.05.

A Random-effect meta-analysis was conducted to account for the possibility that the studies were not estimating the same intervention effect [24]. Heterogeneity was assessed by considering the similarity of point estimates, the overlap of confidence intervals, the context of the results, and the I^2^ statistic in the forest plots [25,26]. To evaluate publication bias and assess the influence of each study, we visually inspected the forest plot and performed sensitivity analyses by excluding individual studies. Funnel plots were not reported, as no meta-analysis included at least 10 trials, which was the recommended threshold for such plots.

### 2.7. Certainty of Evidence Assessment

The certainty of evidence was evaluated using GRADE Evidence Profiles by independent reviewers. Evidence was categorized as “high”, “moderate”, “low”, or “very low” to guide researchers and clinicians in interpreting the significance of the findings. This assessment was based on several key domains, including risk of bias, inconsistency, indirectness, imprecision, and other considerations.

The certainty of evidence was downgraded based on several factors: risk of bias (one level if ≥25% of participants were from studies classified as poor or fair methodological quality, and two levels if ≥50%), inconsistency of results (one or two levels depending on point estimate similarity, confidence interval overlap, I^2^ statistic, and result context), indirectness of evidence (one level for differences in populations, interventions, or comparators), and imprecision (one or two levels for small sample sizes and wide confidence intervals) [25,26].

## 3. Results

**Figure 1:**
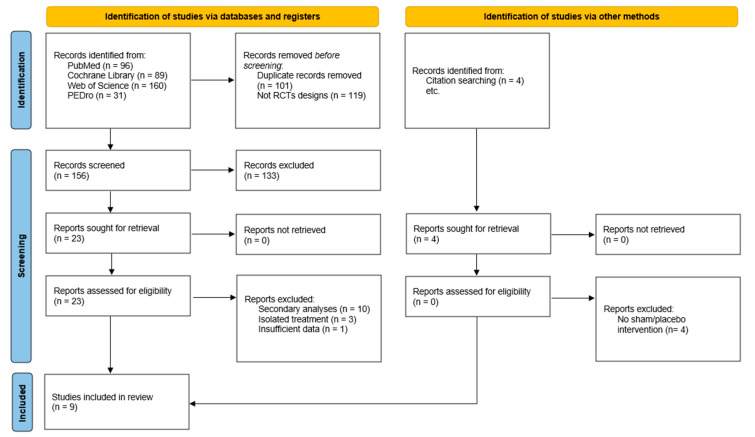
Flowchart diagram of the study.

Nine studies were eventually included in the qualitative synthesis and seven were included in the quantitative synthesis. The secondary analyses from the studies by Licciardone et al. [27,28,29,30,31,32,33,34] and Hansel et al. [35,36] were excluded to avoid data duplication, three studies were excluded for applying a single osteopathic technique without mentioning the holistic diagnosis of the patients [37,38,39], as well as another study that did not provide separate data for patients with NP and LBP [40] (Appendix B). The selection process is shown in the PRISMA flowchart diagram (Figure 1).

### 3.1. Characteristics of the Included Studies

Nine RCTs were included, two comprising 26 patients with NP and seven comprising 1281 patients with LBP.

The studies included patients with non-specific NP [41,42], non-specific LBP [43,44,45,46,47], and pregnant women with LBP [48,49]. The sociodemographic and clinical characteristics of the participants of each study are shown in Table 1.

The interventions applied varied widely, but all were based on individual diagnoses of somatic dysfunctions. Each study pragmatically employed a range of OMTs, combining articular, myofascial, cranial, and/or visceral techniques. Regarding the frequency and duration of the interventions, the most common treatment schedule was one session every one to two weeks, with the intervention duration typically ranging from eight to twelve weeks. A detailed description of the interventions used in each study is provided in Table 2.

The outcome variables were pain intensity, disability, and quality of life. The instruments used to measure these outcome variables in each study are listed in Table 1. Pain intensity was assessed using either the visual analog scale (VAS) or the numeric rating scale (NRS). Disability was evaluated with instruments such as the Neck Disability Index (NDI), Quebec Back Pain Disability Index (QBPDI), Oswestry Disability Index (ODI), and Roland Morris Disability Questionnaire (RMDQ). Quality of life was measured using the Short Form-12 or -36 Health Surveys (SF-12, SF-36). All studies measured these outcome variables both at baseline and after the intervention.

### 3.2. Methodological Quality

The assessment of methodological quality showed that three studies scored four or five points on the PEDro scale and were classified as having fair methodological quality [42,44,45]. Six studies scored between six to eight points and were rated as having good methodological quality [43,49]. One of the most common methodological flaws was that no study blinded the therapist administering the intervention, which is difficult in studies of manual therapy. Additionally, most studies failed to blind participants and did not perform an intention-to-treat analysis. The PEDro scale scores for all studies are shown in Table 3.

### 3.3. Synthesis of Results

#### 3.3.1. Pain Intensity

**Figure 2:**
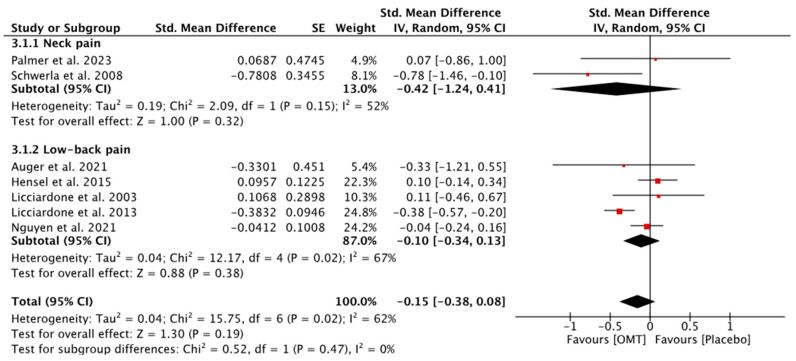
Forest plot for pain intensity in NP and LBP [41,42,43,44,46,47,49].

In the qualitative synthesis, eight out of nine studies assessing pain intensity did not report statistically significant differences between both groups. Only one study achieved statistically significant improvements in favor of the OMT group [47]. The study conducted by Schwerla et al. measured average pain, worst pain, and best pain, and found statistically significant differences in favor of the OMT group only for average pain [41]. The quantitative analysis (meta-analysis) showed that OMT is not statistically superior to sham or placebo interventions in improving pain intensity (Standardized Mean Difference [SMD] = −0.15; −0.38, 0.08; seven studies; 1173 patients), neither for NP (SMD = −0.42; −1.24, 0.41; two studies; 55 patients) nor for LBP (SMD = −0.10; −0.34, 0.08; five studies; 1118 patients) (Figure 2). The certainty of the evidence was downgraded to very low for patients with NP and to low for patients with LBP (Appendix C).

#### 3.3.2. Disability

**Figure 3:**
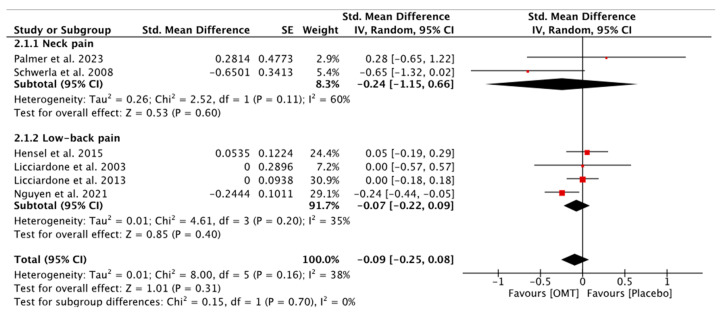
Forest plot for disability in NP and LBP [41,42,43,46,47,49].

In the qualitative synthesis, six studies out of seven assessing disability did not report statistically significant differences between both groups. Only the study of Nguyen et al. showed statistically significant differences in favor of the OMT group for disability [43]. The quantitative analysis (meta-analysis) showed that OMT is not statistically superior to sham or placebo interventions in improving disability (SMD = −0.09; −0.25, 0.08; six studies; 1153 patients), neither for NP (SMD = −0.24; −1–15, 0.66; two studies; 55 patients) nor for LBP (SMD = −0.07; −0.22, 0.09; four studies; 1098 patients) (Figure 3). The certainty of the evidence was downgraded to very low for patients with NP and moderate for patients with LBP (Appendix C).

#### 3.3.3. Quality of Life

Six studies assessed quality of life. Two of them used the questionnaire SF-36 and found no statistically significant differences between both groups [46,47]. Three of them assessed only the physical and mental health subscales of the SF-12, reporting no statistically significant differences between both groups [42,43,44]. Only one study assessed the subscale of bodily pain of the SF-36 and achieved statistically significant differences in favor of the OMT group [41]. No meta-analysis was conducted due to insufficient data in the included studies.

## 4. Discussion

The aim of this systematic review and meta-analysis was to determine whether OMTs for somatic dysfunctions are more effective than sham or placebo interventions in improving pain intensity, disability, and quality of life in patients with NP or LBP. The qualitative synthesis showed that most studies found no statistically significant differences between both interventions, and the quantitative synthesis supports this finding.

The methodological quality of the included clinical trials was mixed. All of the scores ranged from fair to good quality. The most common methodological flaw was the lack of blinding therapists, which is difficult in manual therapy studies. Thus, these studies are inevitably open to bias. The second most common methodological flaw was the lack of intention-to-treat analysis.

The results of our systematic review and meta-analysis are contrary to those found in previous reviews. However, those reviews had serious methodological issues [12,13,14,15]. To avoid the methodological biases identified in earlier studies, our study included only clinical trials published after a peer-review process that applied holistic osteopathic interventions based on a pragmatic diagnosis of somatic dysfunctions, compared with a simulated intervention or placebo. On the other hand, our results are in line with previous systematic reviews with meta-analyses concluding that isolated osteopathic interventions, such as visceral osteopathy [16,50,51] or cranial osteopathy [17,52,53,54], have no clinical effects on musculoskeletal pathologies. Yet, previous studies have shown that OMT is more effective than no intervention in patients with NP [55,56] or LBP [12,57,58]; however, when compared to other interventions, the effects appear to be smaller. These results are likely due to placebo rather than the specific effects of OMT. In other words, the application of real OMT and sham OMT may produce the same or similar neurophysiological effects in the patients, which explains the lack of statistically significant changes between both groups [59,60].

The studies included were based on individualized osteopathic diagnoses through manual palpation of various somatic dysfunctions. Several authors have demonstrated that these are unreliable [51,54,60,61]. In the case of cranial osteopathy, it has been demonstrated that the manual detection of the primary respiratory mechanism or movement restrictions in the skull are unreliable [54]. As for visceral osteopathy, it has been shown that visceral movement impairment is not related to the origin of pathologies, and the palpation of the movement or tension of the viscera is unreliable [51]. Regarding myofascial release, only post-surgical or post-traumatic studies have demonstrated the presence of fascial restrictions or adhesions, and the force required to modify these tissues cannot be achieved manually [62]. Other studies have raised concerns about the reliability of manual palpation for detecting hypomobile segments in the spine. Therapists often misidentify vertebral levels, typically deviating by at least one segment, which increases the risk of misclassification and reduces the diagnostic validity of these methods [63]. It follows that the individualized diagnosis of somatic dysfunctions presents serious limitations in terms of validity and reliability.

Our review has several limitations. Firstly, the searches were conducted in the most relevant databases; however, some studies not indexed in these sources may have been missed. Secondly, the diverse NP and LBP diagnosis, as well as the lack of data reported by some studies, complicates the interpretation of the results and may weaken our conclusion. Thirdly, the primary studies pragmatically applied interventions based on diagnoses of various somatic dysfunctions, resulting in a high degree of heterogeneity among the treatments applied.

## 5. Conclusions

The findings of this systematic review and meta-analysis reveal that OMT is not superior to sham or placebo interventions for improving pain intensity, disability, and quality of life in patients with NP or LBP.

## Figures and Tables

**Table 1:** Characteristics of the included studies and main results.

	Participants		Intervention		Outcome (Tool)	Main Results
Author (Year)	Mean Age (SD)	Diagnosis	OMT Group	Sham/Placebo Group		
Palmer et al., 2023 [42]	OMT:25.4 (2.4) Sham:25.0 (1.8)	NSNP	OMT (*n* = 10)	Light touch (*n* = 8)	Pain (VAS)	ND
					Disability (NDI)	ND
					QoL (SF-12)	
					- Physical	ND
					- Mental	ND
Schwerla et al., 2008 [41]	OMT:41.5 (6.1) Sham:44.8 (9.4)	NSNP	OMT (*n* = 21)	Placebo ultrasound (*n* = 16)	Pain (NRS)	
					- Actual pain	ND
					- Average pain	↑
					- Worst pain	ND
					QoL (SF-36)	
					- Bodily pain	↑
Auger et al., 2021 [44]	OMT:25.9 (2.5) Sham:25.3 (1.6)	NSLBP	OMT (*n* = 10)	Light touch (*n* = 10)	Pain (VAS)	ND
					Disability (ODI)	ND
					QoL (SF-12)	
					- Physical	ND
					- Mental	ND
Nguyen et al., 2021 [42]	OMT:48.3 (11.9) Sham:47.5 (10.6)	NSLBP	OMT (*n* = 164)	Light touch (*n* = 159)	Pain (NRS)	ND
					Disability (QBPDI)	↑
					QoL (SF-12)	
					- Physical	ND
					- Mental	ND
Hensel et al., 2015 [49]	OMT:23.9 (4.1) Sham:24.1 (4.1)	Pregnant women with LBP	OMT (*n* = 136)	Placebo ultrasound (*n* = 133)	Pain (VAS)	
					- Actual pain	ND
					- Average pain	ND
					- Best pain	ND
					- Worst pain	ND
					Disability (RMDQ)	ND
Licciardone et al., 2013 [47]	OMT:41 (29–51) Sham:40 (29–50)	NSCLBP	OMT (*n* = 230)	Light touch (*n* = 225)	Pain (VAS)	↑
					Disability (RMDQ)	ND
					QoL (SF-36)	ND
Licciardone et al., 2010 [48]	OMT:23.8 (5.5) Sham:23.7 (4.4)	Pregnant women with LBP	OMT (*n* = 48)	Placebo ultrasound (*n* = 47)	Pain (VAS)	ND
					Disability (RMDQ)	ND
Licciardone et al., 2003 [46]	OMT:49 (12) Sham:52 (12)	NSCLBP	OMT (*n* = 32)	Light touch and sham OMT (*n* = 19)	Pain (VAS)	ND
					Disability (RMDQ)	ND
					QoL (SF-36)	ND
Gibson et al., 1985 [45]	OMT:34 (14) Sham:40 (14)	NSLBP	OMT (*n* = 35)	Placebo short-wave diathermy (*n* = 33)	Pain (VAS)	
					- Daytime pain	ND
					- Nocturnal pain	ND

OMT: Osteopathic manipulative treatment; NSNP: non-specific neck pain; NSLBP; non-specific low-back pain; LBP: low-back pain; NSCLBP: non-specific chronic low-back pain; VAS: visual analog scale; NRS: numerical rating scale; NDI: neck disability index; ODI: oswestry disability index; QBPDI: Quebec back pain disability index; RMDQ: Roland Morris disability questionnaire; QoL: quality of life; SF-12: short-form health survey; SF-36: short-form health survey; ND: no statistical differences; ↑: statistically significant differences in favor to the OMT group.

**Table 2:** Characteristics of the interventions.

	Intervention				
Author (Year)	OMT Group	Sham/Placebo Group	Session Duration	Frequency	Total Number of Sessions (Weeks)
Palmer et al., 2023 [42]	- Suboccipital release - Cervical contralateral traction - Upper thoracic spine unilateral soft tissue pressure - Thoracic inlet/outlet myofascial release - Atlanto–occipital and atlanto–axial, and C2-7 somatic dysfunction muscle energy - T1-4 somatic dysfunction muscle energy technique - First-rib elevation dysfunction articulation - Submandibular myofascial release - Counterstrain technique	Light touch	OMT: NR Sham: 5 min	3 sessions/week	9 (3 weeks)
Schwerla et al., 2008 [41]	- HVLA techniques - Muscle energy techniques - Myofascial release techniques - Balanced ligamentous tension - Visceral techniques - Cranial techniques	Placebo ultrasound	OMT: 45 m Sham: 12 m	OMT: 1 session every 12–20 days Sham 1 session every 4 to 10 days	9 (NR)
Auger et al., 2021 [44]	- Regional thoracic myofascial release - Lumbar soft tissue - Psoas, piriformis, quadratus lumborum counterstrain - Lumbosacral myofascial release - Sacrum balanced ligamentous tension - Lumbar muscle energy	Light touch	NR	3 sessions/week	9 (3 weeks)
Nguyen et al., 2021 [43]	- Articular techniques - HVLA techniques - Balanced ligamentous tension technique - Cranial techniques - Counterstain techniques - Muscle energy techniques - Myofascial release - Visceral techniques	Light touch	45 m	1 session each 2 weeks	6 (12 weeks)
Hensel et al., 2015 [49]	- Thoracic articulation - Cervical soft tissue - Atlanto–occipital decompression - Thoracic inlet myofascial release - Scapulothoracic soft tissue - Lumbar soft tissue - Diaphragm myofascial release - Sacro-iliac articulation - Pubic symphysis decompression - Frog leg sacral release - Compression of the fourth ventricle	Placebo ultrasound	NR	Sessions at weeks 30, 32, 34, 36, 37, 38, 39	7 (10 weeks)
Licciardone et al., 2013 [47]	- HVLA techniques - Moderate-velocity, moderate-amplitude thrusts - Soft tissue stretching - Myofascial release	Light touch	15 m	Sessions at weeks 0, 1, 2, 4, 6, and 8	16 (8 weeks)
Licciardone et al., 2010 [48]	- Range-of-motion mobilization - Muscle energy - Myofascial release - Soft tissue techniques	Placebo ultrasound	30 m	Sessions at weeks 30, 32, 34, 36, 37, 38, 39	7 (10 weeks)
Licciardone et al., 2003 [46]	- Myofascial release - Strain–counterstrain - Muscle energy - Soft tissue - HVLA - Cranial–sacral	Light touch and sham OMT	15–30 m	Sessions at weeks 1, 2, and then monthly	7 (24 weeks)
Gibson et al., 1985 [45]	- Soft tissue manipulation - Passive articulation of stiff spinal segments - HVLA	Placebo short-wave diathermy	NR	1 session/week	4 (4 weeks)

OMT: osteopathic manipulative treatment; NR: not reported.

**Table 3:** PEDro scale scores.

Author	Items											Total
	1	2	3	4	5	6	7	8	9	10	11	
**Neck pain**												
Palmer et al., 2023 [42]	Y	Y	N	Y	N	N	Y	Y	N	N	N	4/10
Schwerla et al., 2008 [41]	Y	Y	Y	Y	Y	N	N	Y	N	Y	Y	7/10
**Low-back pain**												
Auger et al., 2021 [44]	Y	Y	N	Y	Y	N	N	Y	N	N	N	4/10
Nguyen et al., 2021 [43]	Y	Y	Y	Y	Y	N	N	N	N	Y	Y	6/10
Hensel et al., 2015 [49]	Y	Y	Y	Y	Y	N	N	N	Y	Y	Y	7/10
Licciardone et al., 2013 [47]	Y	Y	Y	Y	Y	N	Y	Y	Y	Y	N	8/10
Licciardone et al., 2010 [48]	Y	Y	Y	Y	N	N	Y	Y	Y	Y	Y	8/10
Licciardone et al., 2003 [46]	Y	Y	Y	Y	Y	N	Y	Y	N	Y	Y	8/10
Gibson et al., 1985 [45]	Y	Y	N	Y	N	N	Y	Y	N	N	Y	5/10

1, eligibility criteria; 2, random allocation; 3, concealed allocation; 4, similarity at baseline; 5, blinding of participants; 6, blinding of therapists; 7, blinding of assessors; 8, measures of at least one key outcome from at least 85% of participants initially allocated to groups; 9, intention to treat analysis; 10, between-group comparison; 11, point measures and measures of variability. 1 = Yes (1 point), 0 = No (0 point), maximum score = 10 (criterion 1 is not included in scores).

## Appendix A. Detailed Search Strategy According to the PRISMA Model

**Search Terms**

**Population**	**Intervention**	**Comparator**	**Study**
Back pain	Osteopathic manipulation	Sham	Clinical trial
Low-back pain	Osteopathic medicine	Placebo	Controlled clinical trial
Sciatica	Osteopathic treatment	Simulated	Randomized controlled trial
Low-back ache	Osteopathic intervention		Trial
Mechanical low-back pain	Osteopathic manipulative treatment		
Lumbago			
Lower back pain			
Low-back ache			
Low-back ache			
Postural low-back pain			
Neck pain			
Chronic neck pain			
Mechanical neck pain			
Non-specific neck pain			
Non-specific chronic neck pain			

**PUBMED**
  (((manipulation, osteopathic[MeSH Terms] OR medicine, osteopathic[MeSH Terms] OR “osteopathic medicine” OR “osteopathic treatment” OR “osteopathic manipulation” OR “osteopathic intervention” OR “osteopathic manipulative treatment” OR osteopath*) AND (neck pain[MeSH Terms] OR “neck pain” OR “chronic neck pain” OR ·”mechanical neck pain” OR “non-specific neck pain” OR “non-specific chronic neck pain” OR low back pain[MeSH Terms] OR back pain[MeSH Terms] OR sciatica[MeSH Terms] OR low back ache[MeSH Terms] OR mechanical low back pain[MeSH Terms] OR “back pain” OR “low back pain” OR sciatica OR lumbago OR “lower back pain” OR “low back ache” OR low backache OR “postural low back pain” OR “mechanical low back pain”)) AND (placebo OR sham OR simulated)))
  Results: 96
  Data: 29 September 2024

**PEDro**
  osteopathic OR osteopathy
  Results: 31
  Data: 29 September 2024

**Cochrane Library**
  ((manipulation, osteopathic OR medicine, osteopathic OR “osteopathic medicine” OR “osteopathic treatment” OR “osteopathic manipulation” OR “osteopathic intervention” OR “osteopathic manipulative treatment” OR osteopath*) AND (neck pain OR “neck pain” OR “chronic neck pain” OR ·”mechanical neck pain” OR “non-specific neck pain” OR “non-specific chronic neck pain” OR low back pain OR back pain OR sciatica OR low back ache OR mechanical low back pain OR “back pain” OR “low back pain” OR sciatica OR lumbago OR “lower back pain” OR “low back ache” OR low backache OR “postural low back pain” OR “mechanical low back pain”) AND (placebo OR sham OR simulated))
  Results: 89
  Data: 29 September 2024

**Web of Science**
  (manipulation, osteopathic OR medicine, osteopathic OR “osteopathic medicine” OR “osteopathic treatment” OR “osteopathic manipulation” OR “osteopathic intervention” OR “osteopathic manipulative treatment” OR osteopath*) (Topic) and (neck pain OR “neck pain” OR “chronic neck pain” OR ·”mechanical neck pain” OR “non-specific neck pain” OR “non-specific chronic neck pain” OR low back pain OR back pain OR sciatica OR low back ache OR mechanical low back pain OR “back pain” OR “low back pain” OR sciatica OR lumbago OR “lower back pain” OR “low back ache” OR low backache OR “postural low back pain” OR “mechanical low back pain”) (Topic) and (placebo OR sham OR simulated) (Topic)
  Results: 160
  Data: 29 September 2024

## Appendix B. Excluded Studies

**Author**	**Reason for Exclusion**
Licciardone, J.C.; Aryal, S. Clinical Response and Relapse in Patients with Chronic Low Back Pain Following Osteopathic Manual Treatment: Results from the OSTEOPATHIC Trial. *Man Ther* 2014, *19*, 541–548, https://doi.org/10.1016/j.math.2014.05.012. [27] Licciardone, J.C.; Gatchel, R.J.; Aryal, S. Targeting Patient Subgroups with Chronic Low Back Pain for Osteopathic Manipulative Treatment: Responder Analyses from a Randomized Controlled Trial. *Journal of the American Osteopathic Association* 2016, *116*, 156–168, https://doi.org/10.7556/jaoa.2016.032. [28] Licciardone, J.C.; Gatchel, R.J.; Aryal, S. Recovery from Chronic Low Back Pain after Osteopathic Manipulative Treatment: A Randomized Controlled Trial. *Journal of the American Osteopathic Association* 2016, *116*, 144–155, https://doi.org/10.7556/jaoa.2016.031. [29] Licciardone, J.C.; Kearns, C.M.; Crow, W.T. Changes in Biomechanical Dysfunction and Low Back Pain Reduction with Osteopathic Manual Treatment: Results from the OSTEOPATHIC Trial. *Man Ther* 2014, *19*, 324–330, https://doi.org/10.1016/j.math.2014.03.004. [30] Licciardone, J.C.; Kearns, C.M.; Minotti, D.E. Outcomes of Osteopathic Manual Treatment for Chronic Low Back Pain According to Baseline Pain Severity: Results from the OSTEOPATHIC Trial. *Man Ther* 2013, *18*, 533–540, https://doi.org/10.1016/j.math.2013.05.006. [31] Licciardone, J.C.; Kearns, C.M.; Hodge, L.M.; Bergamini, M.V.W. Associations of Cytokine Concentrations With Key Osteopathic Lesions and Clinical Outcomes in Patients With Nonspecific Chronic Low Back Pain: Results From the OSTEOPATHIC Trial. *Journal of American Osteopathic Association* 2012, *112*, 596–605. [32] Licciardone, J.C.; Kearns, C.M. Somatic Dysfunction and Its Association With Chronic Low Back Pain, Back-Specific Functioning, and General Health: Results From the OSTEOPATHIC Trial. *Journal of American Osteopathic Association* 2012, *112*, 420–428. [33] Licciardone, J.C.; Gatchel, R.J.; Kearns, C.M.; Minotti, D.E. Depression, Somatization, and Somatic Dysfunction in Patients with Nonspecific Chronic Low Back Pain: Results from the OSTEOPATHIC Trial. *J Am Osteopath Assoc* 2012, *112*, 783–791. [34]	Secondary analyses of the OSTEOPATHIC trial conducted by Licciardone et al. excluded to avoid data duplication.
Hensel, K.L.; Pacchia, C.F.; Smith, M.L. Acute Improvement in Hemodynamic Control after Osteopathic Manipulative Treatment in the Third Trimester of Pregnancy. *Complement Ther Med* 2013, *21*, 618–626, https://doi.org/10.1016/j.ctim.2013.08.008. [35] Hensel, K.L.; Roane, B.M.; Chaphekar, A.V.; Smith-Barbaro, P. PROMOTE Study: Safety of Osteopathic Manipulative Treatment during the Third Trimester by Labor and Delivery Outcomes. *Journal of the American Osteopathic Association* 2016, *116*, 698–703, https://doi.org/10.7556/jaoa.2016.140. [36]	Secondary analyses of the PROMOTE study conducted by Hensel et al. excluded to avoid data duplication.
Ajimsha, M.S.; Daniel, B.; Chithra, S. Effectiveness of Myofascial Release in the Management of Chronic Low Back Pain in Nursing Professionals. *J Bodyw Mov Ther* 2014, *18*, 273–281, https://doi.org/10.1016/j.jbmt.2013.05.007. [37]	Application of a single osteopathic technique without conducting a holistic assessment of the patients’ somatic dysfunctions. Therefore, it does not meet the eligibility criteria for this study.
Klein, R.; Bareis, A.; Schneider, A.; Linde, K. Strain-Counterstrain to Treat Restrictions of the Mobility of the Cervical Spine in Patients with Neck Pain-A Sham-Controlled Randomized Trial. *Complement Ther Med* 2013, *21*, 1–7, https://doi.org/10.1016/j.ctim.2012.11.003. [38]	Application of a single osteopathic technique without conducting a holistic assessment of the patients’ somatic dysfunctions. Therefore, it does not meet the eligibility criteria for this study.
Guthrie, R.A.; Bedford, D.; Ralph Martin, T.H. Effect of Pressure Applied to the Upper Thoracic (Placebo) versus Lumbar Areas (Osteopath Ic Manipula Tive Treatment) for Inhibition of Lumbar Myalgia during Labor. *J Am Osteopath Assoc* 1982, *82*, 247–251. [39]	Application of a single osteopathic technique without conducting a holistic assessment of the patients’ somatic dysfunctions. Therefore, it does not meet the eligibility criteria for this study.
Williams, N.H.; Wilkinson, C.; Russell, I.; Edwards, R.T.; Hibbs, R.; Linck, P.; Muntz, R. Randomized Osteopathic Manipulation Study (ROMANS): Pragmatic Trial for Spinal Pain in Primary Care. Fam Pract 2003, 20, 662–669, https://doi.org/10.1093/fampra/cmg607. [40]	Inclusion of patients with NP and LBP without differentiated data between the two types of patients, which does not allow for its inclusion in either qualitative or quantitative analysis.

## Appendix C. Certainty of Evidence with GRADEPro

**Certainty of Evidence**							**Patients**		**Effect**		**Certainty**
**Nº of Studies**	**Study Design**	**Risk of Bias**	**Inconsistency**	**Indirect-ness Evidence**	**Imprecision**	**Others**	**[OMT]**	**[Placebo]**	**Relative (95% CI)**	**Absolute(95% CI)**	
**Pain intensity (VAS or NPRS) in NP**											
2	RCTs	Serious ^a^	Serious ^b^	Serious ^c^	Very serious ^d^	None	31	24	-	SMD −0.42; (−1.24, 0.41)	⨁◯◯◯ Very low
**Disability (NDI, ODI or RMDQ) in NP**											
2	RCTs	Serious ^a^	Serious ^b^	Serious ^c^	Very serious ^d^	None	31	24	-	SMD −0.24 (−1.15, 0.66)	⨁◯◯◯ Very low
**Pain intensity (VAS or NPRS) in LBP**											
5	RCTs	Not serious	Serious ^b^	Serious ^c^	Not serious	None	588	546	-	SMD −0.10; (−0.38, 0.13)	⨁⨁◯◯ Low
**Disability (NDI, ODI or RMDQ) in LBP**											
4	RCTs	Not serious	Not serious	Serious ^c^	Not serious	None	578	536	-	SMD −0.07 (−0.22, 0.09)	⨁⨁⨁◯ Moderate
CI: confidence interval; SMD: standardized mean difference. Explanations: ^a^ More than 25% of the participants were from studies with poor or fair methodological quality, considering the following aspects: lack of allocation concealment, random allocation and/or sample size calculation, participant and personnel blinding, blinding of outcome assessors. ^b^ I^2^ level was higher than 50%. ^c^ Indirectness was downgraded because the interventions were heterogeneous. ^d^ Population included in each group < 30 participants. High: We are very confident that the true effect is close to the estimate of the effect. Moderate: We are moderately confidence in the effect estimate. The true effect is close to the estimate of the effect, but the result can be different. Low: Confidence in the effect estimate is limited, and the true effect can be substantially different from the estimate of the effect. Very Low: There is little confidence in the effect estimate, and the true effect is likely to be substantially different from the estimated effect.											
